# Utilizing Cooperative Proton–Electron Mixed Conduction Induced via Chemical Dedoping of Self‐Doped Poly(3,4‐ethylenedioxythiophene) Nanofilms for In‐Material Physical Reservoirs

**DOI:** 10.1002/advs.202520270

**Published:** 2025-12-18

**Authors:** Yuya Ishizaki‐Betchaku, Motoaki Onishi, Tomoki Misaka, Mitsuo Hara, Hirokazu Yano, Hidenori Okuzaki, Jun Matsui, Tsuyoshi Hasegawa, Takuya Matsumoto, Hirofumi Tanaka, Shusaku Nagano

**Affiliations:** ^1^ College of Science Rikkyo University 3‐34‐1 Nishi‐Ikebukuro Toshima 171‐8501 Japan; ^2^ Department of Chemistry Graduate School of Science The University of Osaka 1–1 Machikaneyama Toyonaka Osaka 560‐0043 Japan; ^3^ Faculty of Engineering and Design Kagawa University 2217‐20 Hayashicho Takamatsu Japan; ^4^ Organic Materials Research Laboratory Tosoh Corporation 4560 Kaisei‐cho Shunan 746‐8501 Japan; ^5^ Graduate Faculty of Interdisciplinary Research University of Yamanashi 4‐4‐37 Takeda Kofu 400‐8510 Japan; ^6^ Faculty of Science Yamagata University 1‐4‐12 Kojirakawa‐machi Yamagata 990‐8560 Japan; ^7^ Department of Pure and Applied Physics Graduate School of Advanced Science and Engineering Waseda University 3‐4‐1 Okubo Shinjuku Tokyo 169‐8555 Japan; ^8^ Graduate School of Life Science and Systems Engineering Kyushu Institute of Technology 2‐4 Hibikino, Wakamatsu Kitakyushu 808‐0196 Japan; ^9^ Research Center for Neuromorphic AI Hardware Kyushu Institute of Technology 2‐4 Hibikino, Wakamatsu Kitakyushu 808‐0196 Japan

**Keywords:** chemical dedoping, lyotropic liquid crystals, materials networks, mixed conductors, reservoir computing

## Abstract

In this study, an intrinsic ion–electron (hole) coequally conductive state is introduced, induced by de‐doping in self‐doped poly(3,4‐ethylenedioxythiophene) (S‐PEDOT) nanofilms, enhances the performance of in‐material physical reservoirs (PRs) through the cooperative utilization of both carriers. A chemical dedoping modulates the electrical characteristics of the S‐PEDOT PRs. Impedance spectroscopy under controlled relative humidity (RH) conditions reveals that the predominant conducting carriers of the S‐PEDOT nanofilms change systematically depending on the RH. Under low RH, the dominant carriers are holes. At a moderate RH (60–80%), the S‐PEDOT nanofilm exhibits a hole–proton mixed conducting state. A further increase in RH leads to predominantly proton carrier conduction. Moreover, the PR performance of the S‐PEDOT nanofilms is evaluated by wave generation and nonlinear autoregressive moving average (NARMA) tasks. The S‐PEDOT PRs display the best performance at RHs ranging from 60% to 80% (mixed conducting state). Therefore, it is concluded that this high PR performance is attributed to the complex dynamics originating from the cooperative hole–proton mixed conducting state of the S‐PEDOT nanofilms. The results obtain in the present study are the first report of PRs using intrinsically ion–electron mixed conducting states, paving the way for the development of high‐performance material‐based PRs.

## Graphical abstract

This study demonstrates a new concept for high‐performance in‐material physical reservoirs (PRs). An intrinsic and cooperative ion–electron state, induced by chemical dedoping in self‐doped poly(3,4‐ethylenedioxythiophene) (S‐PEDOT) nanofilms, enhances the performance of in‐material PRs. This finding is the first report of in‐material PRs using intrinsically ion–electron mixed conducting states, paving the way for the development of high‐performance material‐based PRs.

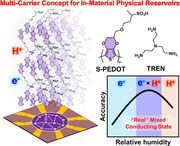

## 1 Introduction

Physical reservoir (PR) computing is an emerging technology that may replace conventional devices based on the von Neumann architecture.^[^ ^1^, ^2^, ^3^, ^4^ ^]^ PRs that mimic neurobiological signal processing mechanisms use the intrinsic nonlinear dynamics of physical systems, including material networks (in‐material PRs) for efficient temporal data processing, which offers significant advantages in energy‐efficient hardware implementations. These PR systems have several requirements, including nonlinearity, high dimensionality, and short‐term memory (fading memory) properties, for efficiently solving time‐dependent computational tasks.^1^ In recent years, various in‐material PR devices based on nanowires,^[^ ^5^, ^6^, ^7^, ^8^, ^9^, ^10^ ^]^ (core–shell) nanoparticles,^[^ ^11^, ^12^, ^13^ ^]^ carbon nanotube (CNT)/polyoxometalate (POM) hybrids,^[^ ^14^, ^15^, ^16^ ^]^ doped Si,^[^ ^17^ ^]^ metal oxide islands,^[^ ^18^ ^]^ organic molecules with nanorod/nanoparticles,^[^ ^19^ ^]^ biomembranes,^[^ ^20^ ^]^ ferroelectric materials,^[^ ^21^ ^]^ liquid crystal molecules,^[^ ^22^ ^]^ and conducting polymers^[^ ^23^, ^24^, ^25^ ^]^ have been reported using individual nonlinear dynamics such as atomic switching, reduction–oxidation (redox) reactions, Coulomb blockade, potential landscapes, ion diffusion, molecular vibration dynamics, memcapacitor characteristics, and polarization changes. We also recently investigated nonlinear elements using different approaches, such as the use of resistive switches,^[^ ^26^, ^27^ ^]^ conducting polymer Langmuir–Blodgett films,^[^ ^28^ ^]^ and fluidic ionic diodes.^[^ ^29^ ^]^ However, most reported PR devices are limited to adopting single carriers (e.g., electrons (holes) or ions) for solving reservoir computing tasks although multi‐carriers are utilized cooperatively in the human body for the transport of ions (e.g., H^+^, Na^+^, K^+^, Ca^+^)^[^ ^30^ ^]^ through biological membranes and for electron transport via redox reactions (e.g., cytochrome c^[^ ^31^ ^]^ and ferritin^[^ ^32^ ^]^). Compared with a single carrier, multiple carriers can potentially provide more complex nonlinear dynamics in a system designed for material‐based PR computing.

Organic mixed ionic electronic conducting (OMIEC) polymers are (semi)conducting polymers that have multi‐carrier systems propagating electron and ion species.^[^ ^33^, ^34^ ^]^ Their applications have rapidly expanded to include supercapacitors,^[^ ^35^, ^36^ ^]^ organic electrodes for batteries,^[^ ^37^, ^38^, ^39^, ^40^ ^]^ actuators,^[^ ^41^ ^]^ light‐emitting devices,^[^ ^42^, ^43^ ^]^ chemical sensors/biosensors,^[^ ^44^ ^]^ ion pumps,^[^ ^45^ ^]^ organic electrochemical transistors,^[^ ^46^, ^47^ ^]^ and neuromorphic devices.^[^ ^48^, ^49^ ^]^ The focus of OMIEC devices is on ion–electron interactions, such as perturbation to the main chain electronic state due to ion diffusion and the conversion of carriers from electrons to ions. OMIEC polymers change the conducting properties of the main chain by doping and de‐doping via the approach and departure of ion species or protonation and deprotonation. Therefore, ion diffusion (conduction) and electronic signals are treated in separate events. In general, for OMIEC polymers, the dominant conduction carrier is an electron (hole) because the mobility of electrons is much greater than that of ions by ≈2–10 orders of magnitude, depending on the type of OMIEC polymer.^[^ ^50^, ^51^, ^52^, ^53^, ^54^ ^]^ To use multi‐carriers simultaneously and effectively, the conductivities (carrier mobilities × carrier densities) of electrons and ions must be of comparable order.

**Figure 1:**
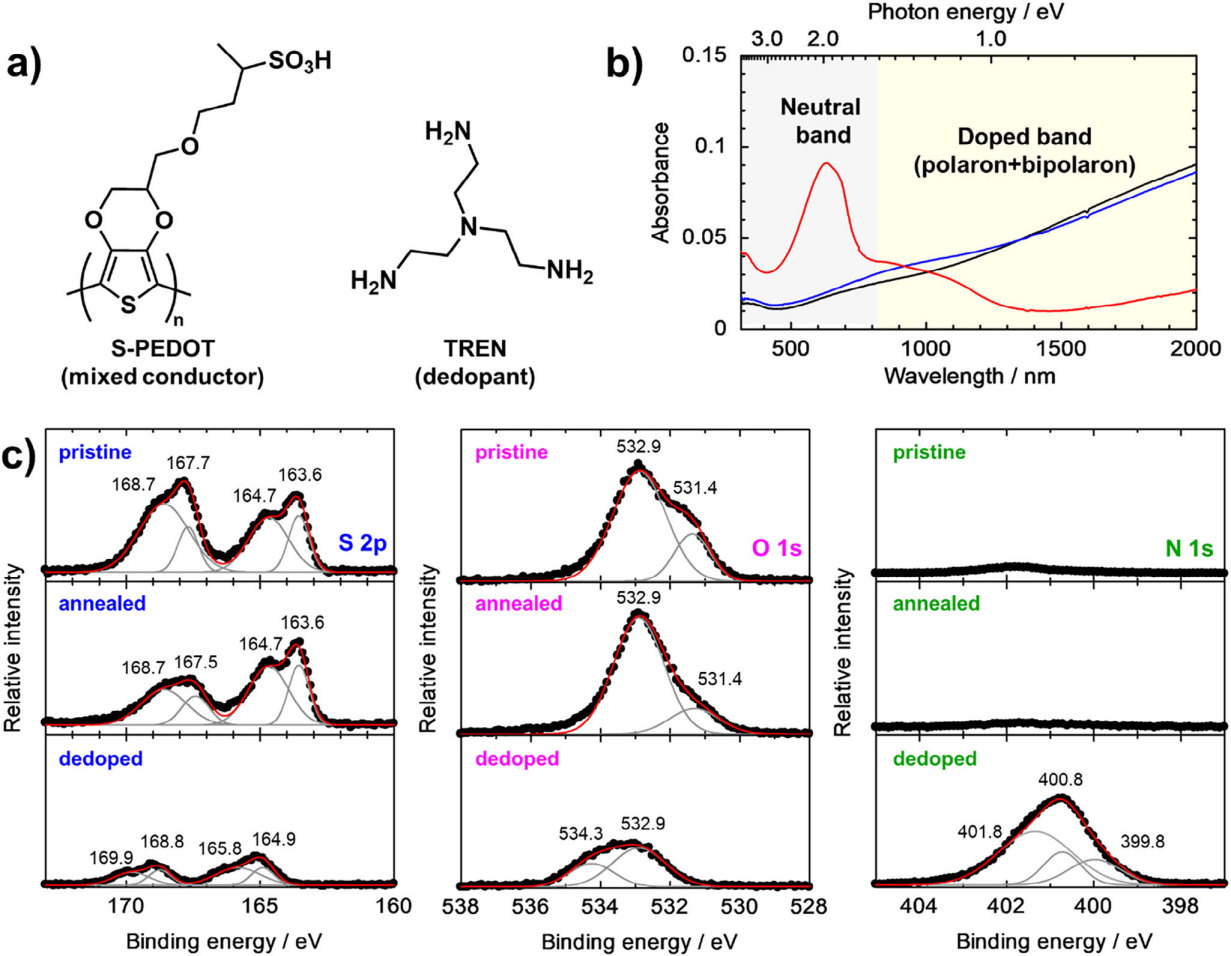
a) Chemical structures of S‐PEDOT (mixed conducting polymers) and TREN (chemical dedopants). b) UV–vis–NIR absorption spectra of 0.25 wt.% S‐PEDOT spin‐cast films before (black) and after annealing under vacuum at 200 °C for 30 min (blue), and after subsequent dedoping by TREN (red). c) S 2p (blue), O 1s (pink), and N 1s (green) XPS spectra of the S‐PEDOT spin‐cast films before (top) and after annealing (middle), and after subsequent dedoping by TREN (bottom). The experimental data (dots) were deconvoluted (gray lines) and fitted (red lines) using multiple Gaussian distribution functions.

We recently reported that self‐doped poly(3,4‐ethylenedioxythiophene) (S‐PEDOT, **Figure** **1**a)^[^ ^55^ ^]^ with a defined chemical structure exhibits a fully water‐soluble nature and high electrical conductivity (1000 S cm^−1^) without external dopant molecules, differing from conventional PEDOT:poly(4‐styrenesulfonate) (PSS) colloidal systems.^[^ ^56^, ^57^ ^]^ The conjugated backbones of S‐PEDOT are doped by protonation from sulfonated groups in the side chains,^[^ ^58^ ^]^ and a well‐ordered lamellar structure is organized by the nanosegregation of *π*‐stacked backbones and ionized side chains. Therefore, S‐PEDOT behaves as a mixed conductor, distinctly forming parallel paths for ion (proton) and electron (hole) carriers at the mesoscale. Moreover, the doping level and electrical conductivity of such doped conducting polymers can be modulated by reacting with an electron donor and a Brønsted–Lowry base (e.g., monoamine or polyamines) by chemical dedoping processes.^[^ ^59^ ^]^ Thus, the intrinsic mixed conduction (multi‐carrier) states provide a new class of materials for in‐material PRs.

We proposed the utilization of the mixed conducting states of intrinsic ion–electron carriers in S‐PEDOT for in‐material PRs and demonstrated its efficacy in this study. The electrical conductivity and doping levels of the S‐PEDOT nanofilms were controlled by a chemical dedoping process using a multivalent polyamine (tris(2‐aminoethyl)amine) (TREN, Figure 1a). The electronic state of S‐PEDOT was investigated by ultraviolet–visible–near‐infrared (UV–vis–NIR) absorption and x‐ray photoelectron spectroscopy (XPS) measurements. The nanostructures of the dedoped S‐PEDOT nanofilms were examined by in situ grazing incidence (GI)‐X‐ray scattering (XRS) measurements under controlled relative humidity (RH) conditions. The ion–electron mixed conducting characteristics were carefully explored by current (*I*)–voltage (*V*) and impedance spectroscopy measurements under different RH conditions. The nonlinear dynamics and physical PR performance of the dedoped S‐PEDOT nanofilms were evaluated by time‐domain voltage (*V*)–time (*t*) measurements, wave generation, and nonlinear autoregressive moving average (NARMA) tasks under different RH conditions as benchmark tasks of reservoir computing. This study is the first report of PRs using intrinsically ion–electron mixed conducting states, paving the way for the development of high‐performance material‐based PRs.

## 2 Results and Discussion

### 2.1 Dedoping of S‐PEDOT Nanofilms

UV–vis–NIR absorption measurements were conducted to investigate the dedoping of the S‐PEDOT spin‐cast films. Figure 1b (black) shows the UV–vis–NIR absorption spectra of S‐PEDOT spin‐cast films prepared from 0.25 wt.% S‐PEDOT aqueous solution. As a result, a broad absorption band appeared at ≥ 1000 nm (≤ 1.24 eV) in the NIR regions, which could be attributable to positive polarons and bipolarons of the S‐PEDOT main chains.^[^ ^55^ ^]^ After the film was annealed at 200 °C for 30 min under vacuum to improve water stability (Figure 1b, blue), minor changes appeared in the absorption band in the NIR region. Specifically, the broad absorption band at ≥1400 nm slightly decreased, whereas the absorption band at 600–1300 nm increased, suggesting the dedoping of some of the S‐PEDOT main chains. Afterward, the films were further dedoped by spin‐coating of TREN/methanol solution and subsequent annealing (Figure 1b, red). Results showed that the absorption attributed to the doped state drastically decreased in the NIR region. In contrast, a new strong absorption band appeared at 630 nm (1.97 eV), which was attributed to the neutral states of the S‐PEDOT main chains.^[^ ^55^ ^]^ These results indicated the successful dedoping of the S‐PEDOT films.

To obtain further insights into the dedoping of the S‐PEDOT nanofilms, the chemical compositions of S‐PEDOT films before and after TREN treatment were analyzed using XPS. Figure 1c (blue and pink, top) displays XPS spectra corresponding to the S 2p and O 1s core‐level signals of a pristine S‐PEDOT film. The S 2p signal shows two major contributions from the sulfur atoms in the thiophene rings of the S‐PEDOT main chains (163.6 and 164.7 eV) and those in the sulfonate groups of the side chains (167.7 and 168.7 eV) with a doublet signal appealing due to spin–orbit coupling.^[^ ^59^ ^]^ The O 1s spectra were deconvoluted into two components: the low binding energy was assigned to oxygen atoms in the ethylenedioxy rings (532.9 eV), and the high binding energy was attributed to that in the sulfonate groups of the side chains (531.4 eV), respectively.^[^ ^60^ ^]^ After annealing, the peak area ratio (*A*_S,SO3H_ /*A*_S,PEDOT_), where *A*_S,SO3H_ and *A*_S,PEDOT_ are the peak area for sulfur atoms from –SO_3_H and thiophene rings, respectively, decreased from 1.0 to 0.55 (Figure 1c blue, middle). Additionally, the O 1s peaks for the sulfonate groups of the side chains decreased while maintaining the peak area attributed to the ethylenedioxy rings (Figure 1c pink, middle). These results suggested that partial crosslinking of S‐PEDOT by the degradation of the ─SO_3_H side chains in S‐PEDOT, resulted in water‐resistant S‐PEDOT films (Figure S1, Supporting Information),^[^ ^61^ ^]^ which was consistent to the results of UV–vis–NIR absorption measurements. Such partial crosslinking and the degradation of the ─SO_3_H side chains in S‐PEDOT are also supported by thermogravimetric–differential thermal analysis (TG–DTA, Figure S2a, Supporting Information), differential scanning calorimetry (DSC, Figure S2b, Supporting Information), and Fourier transform near infrared (FT‐IR, Figure S3, Supporting Information) measurements. Next, we chemically dedoped the S‐PEDOT films with TREN. The results revealed that both S 2p signals shifted ≈1 eV to higher binding energy regions, while maintaining the peak area ratio (Figure 1c blue, bottom). These peak shifts were attributed to the dedoped S‐PEDOT main chains and the formation of ion pairs between sulfonate groups in the S‐PEDOT side chains and TREN,^[^ ^62^, ^63^, ^64^ ^]^ respectively. A similar tendency was observed for the O 1s spectra (Figure 1c pink, bottom). The N 1s core‐level signals after dedoping (Figure 1c green, bottom) supported the formation of ion pairs between sulfonate groups and TREN because the signal assigned to protonated amines (─NH_3_^+^, 401.8 eV) was the major component; minor components were attributed to primary amines (─NH_2_, 399.8 eV) and tertiary amines (═N─, 400.8 eV).^[^ ^65^ ^]^ Notably, the very weak signals for the pristine and annealed S‐PEDOT nanofilms could originate from contamination (Figure 1c green, top and middle). Subsequently, we performed electron spin resonance (ESR) measurements of S‐PEDOT films before and after dedoping (Figure S4, Supporting Information). As a result, the ESR intensity increased upon the dedoping processes by TREN. This indicates the transformation from bipolaronic species to the polarons of S‐PEDOT via a single‐electron transfer process. In fact, the obtained EPR spectra displayed a pronounced decrease in the half‐width, indicating the generation of localized radical species through chemical dedoping. Note that the doping/dedoping reaction may involve a complex radical process.^[^ ^59^, ^66^, ^67^ ^]^ From the results mentioned above, the possible mechanism of S‐PEDOT dedoping is as follows (Figure S5, Supporting Information). S‐PEDOT is slightly dedoped by the degradation of partial ─SO_3_H groups in the side chains by thermal annealing. After that, S‐PEDOT is chemically dedoped by proton abstraction from the main chains by TREN. The subsequent single‐electron transfer creates dedoped PEDOT main chains in neutral states. The protonated TREN molecules form an ion pair with the residual ─SO_3_^–^ side chains of S‐PEDOT for charge compensation. The formed ion pair further cross‐links the sulfonated groups of the S‐PEDOT side chains.

We subsequently investigated the surface morphologies of the S‐PEDOT films before and after dedoping. Figure S6a,b (Supporting Information) displays atomic force microscopy (AFM) images of 0.25 wt.% S‐PEDOT films before and after dedoping. The pristine S‐PEDOT film showed a smooth and uniform surface morphology with a low surface roughness (≤ 0.52 nm, Figure S6a, Supporting Information) and an ultrathin film thickness (average film thickness = 10.8 ± 0.4 nm (*n* = 5), Figure S6c, Supporting Information). Moreover, the smooth surface morphology remained after dedoping by TREN (Figure S6b, Supporting Information). This result presented a unique advantage of S‐PEDOT compared to the conventional PEDOT:PSS films, making the formation of thinner films more difficult than those of colloidal particle size.^[^ ^55^, ^56^, ^57^ ^]^ Therefore, we successfully prepared dedoped uniform ultrathin S‐PEDOT nanofilms.

### 2.2 Electrical Characteristics and Nanostructures of the S‐PEDOT Nanofilms

**Figure 2:**
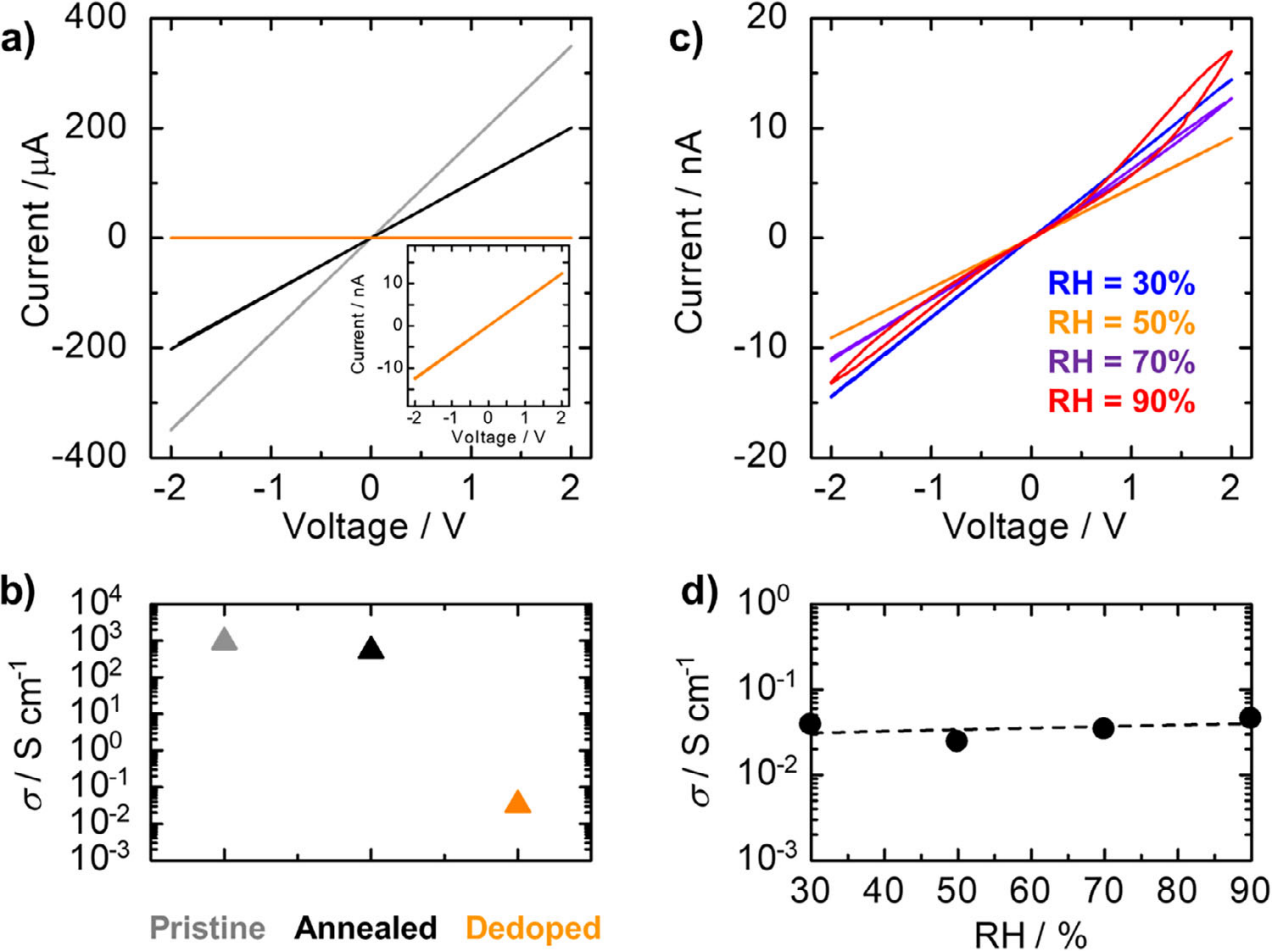
a) *I–V* curves of 0.25 wt.% S‐PEDOT nanofilms at 25 °C at 30% RH before (gray) and after annealing (black) and after subsequent dedoping by TREN (orange). The inset shows magnified *I–V* curves in orange. b) Electrical conductivities calculated from a). c) *I–V* curves of the S‐PEDOT nanofilms under different RH conditions at 25 °C; RH = 30% (blue), 50% (orange), 70% (purple), and 90% (red). d) Electrical conductivity as a function of RH calculated from c).

**Figure** **2**a (gray) shows the *I–V* curve of a pristine S‐PEDOT nanofilm at 25 °C under controlled RH conditions (RH = 30%). The results revealed ohmic conduction with high electrical conductivity (*σ* = 9.3 × 10^2^ S cm^−1^; Figure 2b (gray)) comparable to that of the bulk sample reported previously,^[^ ^55^ ^]^ even with the ultrathin film thickness (≈10 nm). The *σ* values slightly decreased to 5.3 × 10^2^ S cm^−1^ after annealing (Figure 2a,b, black). This decrease in electrical conductivity was attributed to the reduction in the number of hole carriers due to the partial decomposition of sulfonate groups in the S‐PEDOT side chains, as noted from the UV–vis–NIR absorption and XPS measurements. Conversely, the electrical conductivity drastically decreased by four orders of magnitude (*σ* = 3.3 × 10^−2^ S cm^−1^), remaining ohmic conduction without a nonlinear shape after being dedoped through TREN treatments (Figure 2a,b, orange).

To further investigate the electrical characteristics of the S‐PEDOT nanofilms, we conducted *I–V* measurements under different RH conditions (Figure 2c). The *I–V* characteristics of the dedoped S‐PEDOT nanofilms strongly depended on the RH. Under low RH conditions (RH ≤ 50%), the *I–V* curves were ohmic (Figure 2c, blue and orange). In contrast, the *I–V* curves exhibited nonlinearity with hysteresis under high humidity conditions (RH ≥ 70%, Figure 2c, purple and red). These capacitive behaviors with hysteresis suggested the contribution of ionic (proton) conduction under high RH conditions. Considering their molecular structures, the S‐PEDOT main chains behave as hole conductors, and the sulfonate side chains operate not only as dopants for S‐PEDOT but also as proton conductors. Interestingly, the electrical conductivity was almost identical to that under RH conditions (Figure 2d). Note that the current from the contribution of proton conduction in the *I–V* measurement is negligible due to the long enough time delay for each data point (see the details in Methods). In this condition, *I–V* curves exhibit only a direct current attributed to hole conduction of the S‐PEDOT main chains. Therefore, we expected that the holes would dominate electrical conduction under low RH conditions. In contrast, the protons might be dominant under high RH conditions because of water adsorption.

**Figure 3:**
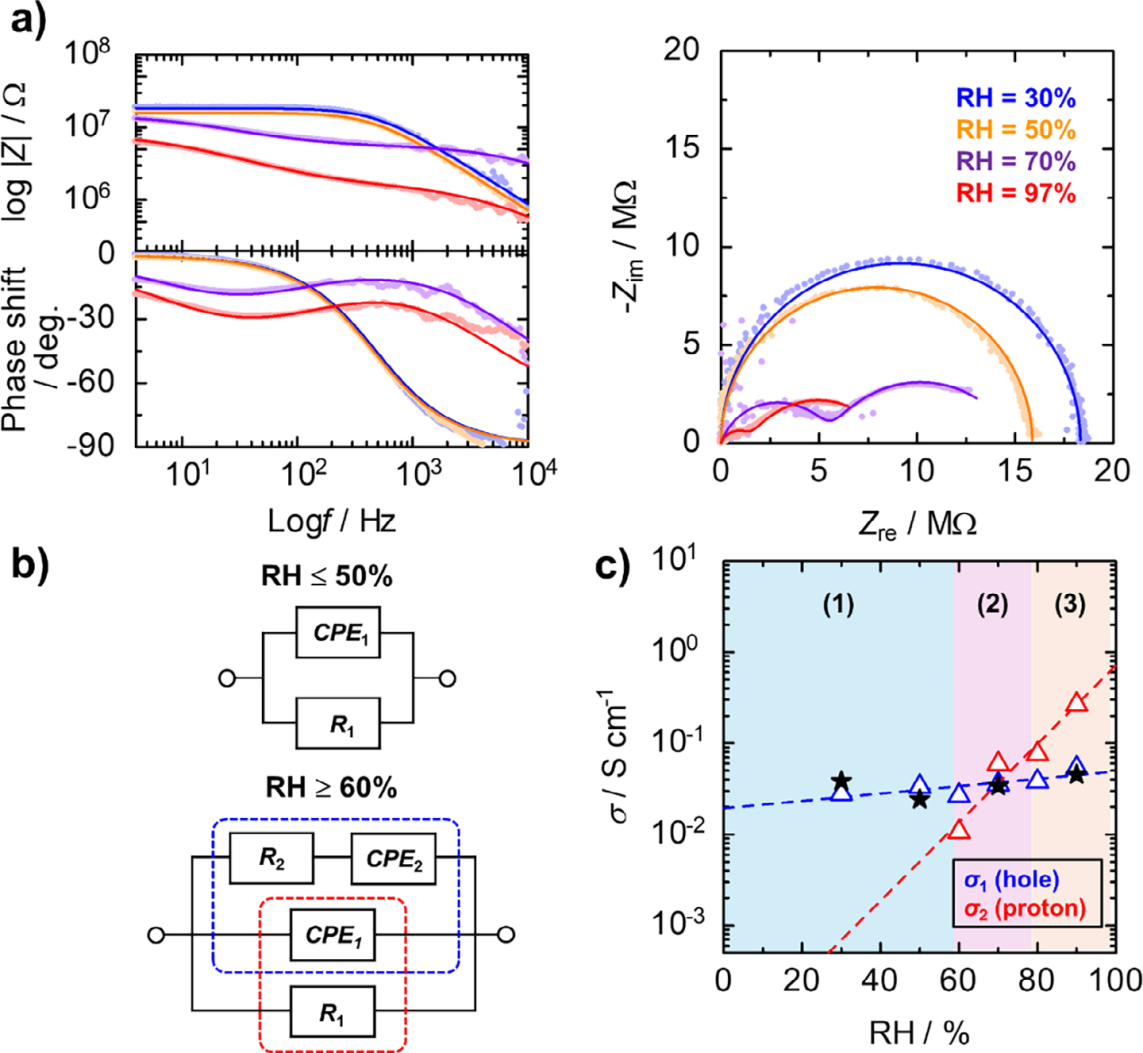
a) Absolute impedance (upper left), Bode phase (lower left), and Nyquist plots (right) of dedoped S‐PEDOT nanofilms at 25 °C under different RH conditions; RH = 30% (blue), 50% (orange), 70% (purple), and 90% (red). The solid lines are fitting lines obtained using an appropriate equivalent circuit model. b) Equivalent circuit models for fitting. c) Plots of two different electrical conductivities (σ_1_ and σ_2_) as a function of the RH values extracted from the fitting in a). Black stars represent the σ values calculated from Figure 2d).

**Figure 4:**
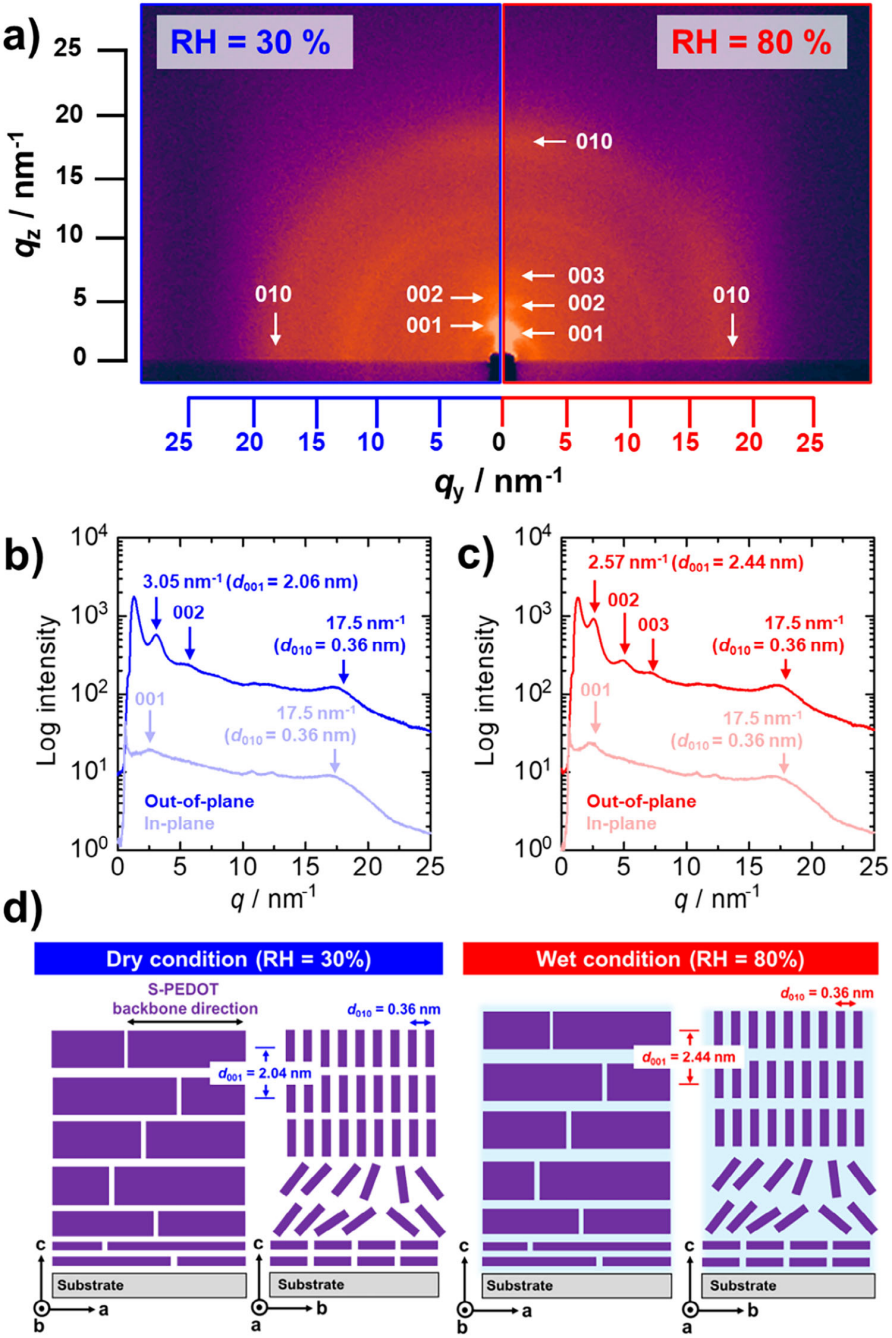
a) In situ 2D GI‐XRS patterns of dedoped S‐PEDOT films measured under 30% (left panel, blue) and 80% (right panel, red) RH conditions. b,c) 1D profiles of the out‐of‐plane (vivid) and in‐plane (pale) directions obtained from a), respectively. d) Schematics of the proposed nanostructures of the dedoped S‐PEDOT nanofilms under dry (left) and wet (right) conditions. For simplicity, TREN is omitted from this image.

For the detailed discussion of the conduction carriers in the S‐PEDOT nanofilms, impedance spectroscopy measurements were performed under controlled temperature and RH conditions. **Figure** **3**a (left) shows absolute impedance (|*Z*|) and Bode phase (*θ*) plots for the dedoped S‐PEDOT nanofilms. The results revealed that a shoulder and a dip of |*Z*| and *θ* appeared in the low frequency (*f*) regions with increasing RH. Additionally, the Nyquist plots in Figure 3a (right), which were prepared by plotting the real part (*Z*_re_) and the imaginary part (*Z*_im_) of the impedance obtained from the absolute impedance and Bode phase plots, exhibited a typical semicircle under low RH conditions (RH ≤ 50%). In contrast, two semicircles were observed under relatively high RH conditions (RH ≥ 60%). These results implied that another component was involved in the system upon increasing the RH value. For quantitative assessment, all experimentally obtained data were fitted using two different equivalent circuit models (Figure 3b)^[^ ^68^, ^69^ ^]^ consisting of resistors (*R*) and a constant phase element (CPE) according to the criterion of the minimum required components to explain the experimentally obtained data. Here, the impedance of the CPE (*Z*_CPE_) component is expressed as *Z*_CPE_ = 1/*Q*(i*ω*)^α^, where *Q* and *α* (0 ≤ *α* ≤ 1) are CPE parameters. When *α* = 1, *Q* acts as a pure capacitor (*C*). The system exhibits deviation from a pure capacitor because of system heterogeneity for *α* < 1. In addition, *ω* represents the angular frequency (*ω* = 2π*f*) and *i* stands for the imaginary number ($i=\sqrt{-1}$). As a result, all the experimental data were well‐explained by the appropriate equivalent circuit models (Figure 3a). All the fitting parameters are summarized in **Table** **1**. From the fitting data, we extracted the electrical resistances (*R*_1_ and *R*_2_) and calculated the electrical conductivities (σ_1_ and σ_2_) for two different components (Figure 3c). Notably, the σ_1_ value was almost independent of the RH values (blue), whereas another component (σ_2_) drastically increased with increasing RH (red). Furthermore, the effective capacitances (*C*_eff,1_ and *C*_eff,2_) calculated from the equation, *C*_eff,n_ = *Q_n_* ^1/^ *^α^R* ^(1–^ *^α^* ^)/^ *^α^*, exhibited similar trends (Table 1). *C*_eff,1_ presented almost constant values regardless of the RH, whereas *C*_eff,2_ increased with increasing RH. The increase in *C*_eff,2_ reflected the increase in system permittivity, suggesting adsorption of water molecules with high permittivity into the films and formation of an electric double layer at the electrode interfaces. Since the σ_1_ value was comparable to the conductivity calculated from the *I–V* measurements in Figure 2d, *R*_1_ could be assigned to the resistance of electronic (hole) conduction. Conversely, *R*_2_, which showed strong RH dependence, could be attributed to proton conduction as mentioned by the increase in *C*_eff,2_. In general, amphiphilic polyelectrolytes with a rigid polymer backbone and water‐soluble side chains (e.g., sulfonated polyimides (SPIs)) exhibit a lyotropic liquid crystalline (LC) nature, and they provide excellent proton conductivity by the formation of lyotropic LC lamellar structures with water nanochannels through water uptake.^[^ ^70^, ^71^ ^]^ In fact, the quartz crystal microbalance (QCM) measurements support water adsorption by the S‐PEDOT nanofilms with increasing RH (Figure S7, Supporting Information). The estimated water content (*λ*_H2O/SO3H_) defined by Equation (1) was calculated as *λ*_H2O/SO3H_ = 13 at the maximum value:

(1) $$\lambda_{\frac{H_{2}O}{SO_{3}H}}=\left(\frac{m-m_{0}}{M_{H_{2}O}}\right)\frac{M_{S-\mathrm{PEDOT}}}{m}$$

where *m* signifies the film mass at each RH condition, *m*_0_ stands for that at the RH = 0% RH, *M*_H2O_ and *M*_S‐PEDOT_ express the molecular weights of a water molecule and an S‐PEDOT monomer unit, respectively. This value was comparable to that of SPI‐based polymer thin films with high proton conductivity as reported in the literature.^[^ ^70^, ^71^ ^]^ Such water uptake affords an increase in the number of conducting carriers due to proton dissociation from the S‐PEDOT side chains upon water adsorption with increasing RH. Moreover, we performed in situ GI‐XRS measurements to reveal the formation of well‐defined lyotropic LC lamellar structures. **Figure** **4**a displays the 2D GI‐XRS patterns of dedoped S‐PEDOT nanofilms under controlled RH conditions. Under dry conditions (RH = 30%), 001 scattering attributed to S‐PEDOT lamellar stacking (*d*_001_ = 2.06 nm) was observed in the out‐of‐plane direction with a small scattering in the in‐plane direction as a minor component (Figure 4b). However, a broad peak assigned to 010 scattering was confirmed in both the out‐of‐plane and in‐plane directions. These results indicated that the dedoped S‐PEDOT nanofilms predominantly adopted an edge‐on orientation as a major component.^[^ ^72^ ^]^ Compared with that under dry conditions, the interlamellar distance of the S‐PEDOT nanofilms increased to *d*_001_ = 2.44 nm with high‐order scattering when the RH was increased to 80% (Figure 4c). These results indicated that the S‐PEDOT nanofilm adopted water uptake by increasing the RH, resulting in the formation of proton‐conducting nanochannels with long‐range order (Figure 4d). Notably, the electrical conductivities of the holes (*σ*_hole_) and protons (*σ*_proton_) were comparable in narrow RH regions from 60% to 80%. The enhanced *σ*_proton_ observed with increasing RH can be attributed to the increase in the number of conducting carriers due to proton dissociation from the S‐PEDOT side chains and the formation of lyotropic LC lamellar domains with long‐range order. These processes effectively reduce the activation energy for proton transport, leading to a significant improvement in proton conductivity under higher RH conditions as reported before.^[^ ^70^, ^71^ ^]^ From the discussion above, we proposed that the predominant conducting carrier of dedoped S‐PEDOT nanofilms changed systematically depending on the RH. Under low RH conditions (RH ≤ 50%), the main conducting carrier was holes (electrons) (Figure 3c, region (1), left). With increasing RH, the S‐PEDOT nanofilm exhibited a mixed conducting state because of its comparable *σ*_hole_ and *σ*_proton_ values under intermediate RH conditions (60% ≤ RH ≤ 80%) (Figure 3c, region (2), middle). This intrinsically mixed conducting state could provide more complex dynamics in the system to improve PR performance. A further increase in RH resulted in a much higher *σ*_proton_ than *σ*_hole_, indicating that the dominant conducting carrier was protons under the high RH conditions (RH > 80%) (Figure 3c, region (3), right). Therefore, we could systematically modulate the dominant conducting carriers by tuning the RH conditions.

**Table 1:** Summary of the fitting parameters extracted from the data in Figure 3a.

RH / %	*R*_1_ / MΩ [*σ*_1_ / S cm^−1^]	*Q*_1_ / pF s*^α^* ^−1^ (*C*_eff,1_ / pF)	*a*_1_ / ‐	*R*_1_ *C*_1,eff_ / ms	*R*_2_ / MΩ (*σ*_2_ / S cm^−1^)	*Q*_2_ / pF s*^α^* ^−1^ (*C*_eff,2_ / pF)	*a*_2_ / ‐	*R*_2_ *C*_2,eff_/ms
30	18.5 (0.028)	18.0 (18.0)	1.0	0.33	– (–)	– (–)	–	–
50	16.0 (0.033)	22.0 (22.0)	1.0	0.35	– (–)	– (–)	–	–
60	20.0 (0.027)	30.0 (25.8)	0.98	0.52	50.0 (0.011)	500 (199)	0.80	9.94
70	15.0 (0.036)	40.0 (8.75)	0.83	0.13	9.0 (0.059)	1500 (305)	0.73	2.75
80	14.0 (0.038)	190 (43.1)	0.80	0.60	7.0 (0.076)	1000 (191)	0.75	1.34
90	10.0 (0.053)	150 (29.5)	0.80	0.30	2.0 (0.27)	5000 (695)	0.70	1.39
98	8.5 (0.063)	200 (40.6)	0.80	0.35	2.0 (0.27)	5000 (695)	0.70	1.39

### 2.3 Nonlinear Dynamics and Physical Reservoir Performance of S‐PEDOT Nanofilms

**Figure 5:**
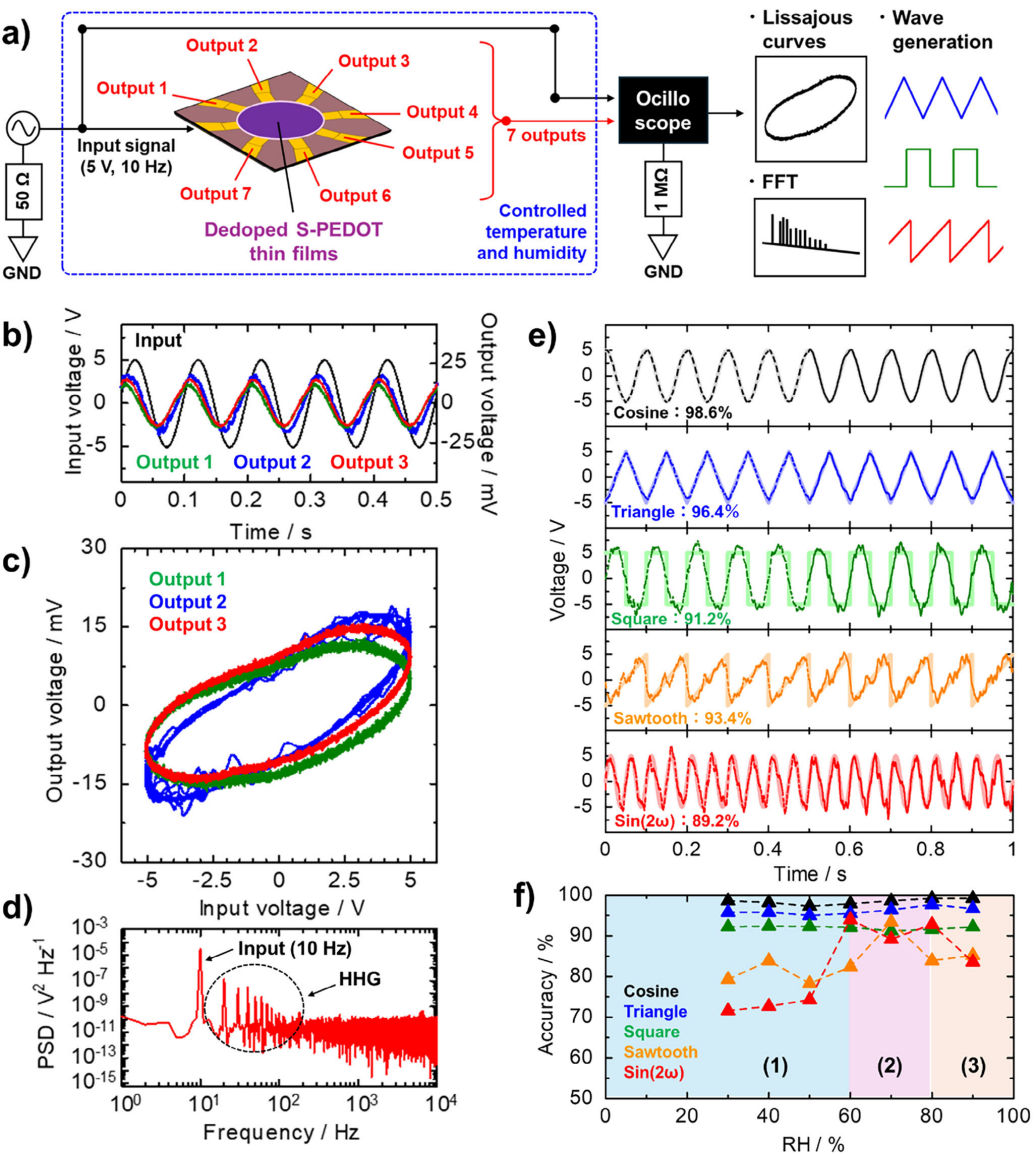
a) Schematics of the experimental setup of *V–t* measurements and wave generation tasks. All the measurements were conducted under controlled RH conditions. b) Time‐domain *V–t* curves of the input (black) and three arbitrarily selected output (colored) signals. c Lissajous profiles (*f* = 10 Hz, *V*_pp_ = 10 V) at 25 °C under 70% RH conditions prepared from b). d) Log–log plots of PSD for an output signal obtained in (b, red). e) Wave generation tasks for cosine (black), triangle (blue), square (green), sawtooth (orange), and sin(2ω) (red) waves reconstructed from seven nonlinearized output waves using the dedoped S‐PEDOT nanofilm device at 25 °C under 70% RH conditions. In these plots, the pale‐colored solid line, dashed lines, and vivid‐colored solid lines represent the target wave, training wave, and predicted wave, respectively. f) Summary of the accuracies calculated from Equation (2) under different RH conditions.

As mentioned in the Introduction, some requirements should be satisfied for PRs, such as nonlinearity, high dimensionality, and fading (short‐term) memory. These requirements for PR devices could be explained by their responses to sinusoidal waves. **Figure** **5**a,b shows the experimental setup and the *V–t* curves of the input sinusoidal waves (black) and their corresponding three representative output signals (colored) for the dedoped S‐PEDOT nanofilms at 25 °C and 70% RH. As a result, different phase shifts and nonlinearly distorted waveforms were observed for each output signal. Furthermore, Lissajous profiles obtained from Figure 5b displayed a distorted ellipse with hysteresis (Figure 5c). This versatile nonlinear transformation of the input wave indicated that the dedoped S‐PEDOT nanofilms provided complex dynamics into the system, which were crucial characteristics for spatiotemporal information processing of PR computing. From the *V–t* curve, the power spectral density (PSD; Figure 5d) was obtained from the output signals in Figure 5b using fast Fourier transform (FFT). The PSD profiles revealed rich higher harmonic generation (HHG; integer multiples of the input sinusoidal wave frequency (10 Hz)) for the output wave, except for a single input frequency (10 Hz); these findings were indicative of the high dimensionality of the S‐PEDOT nanofilms.

Subsequently, waveform generation tasks were conducted as a benchmark task to demonstrate PR performance. Figure 5e shows the results of the waveform generation task with target waveforms (cosine, triangle, square, sawtooth, and sin(2ω)) for dedoped S‐PEDOT nanofilms at 25 °C and 70% RH. A 10 Hz sinusoidal wave (*V*_pp_ = 10 V) was input into the device using a function generator. The five waveforms were generated with ridge regression using all 7 outputs of the device. The output waves were collected over one second using a digital oscilloscope. The first half of the collected time series data were used as training data (Figure 5e, dashed lines), and the last half of the data were used as test data (Figure 5e, solid lines). The accuracy of the results was evaluated via Equation (2):

(2) $$Accuracy\;\left(x,y\right)=1-\frac{\sum_{i=0}^{n-1}{\left(x_{i}-y_{i}\right)}^{2}}{\sum_{i=0}^{n-1}{\left(x_{i}-\bar{x}\right)}^{2}}$$

where *x*_i_, $\bar{x}$, *y*_i_, and *n* are the output, mean of the output, target, and the number of outputs (*n* = 7), respectively. The generated waves reproduced the target waveforms with high accuracy (≈90%) regardless of the waveform (Figure 5e). The high accuracies under humid conditions suggested that S‐PEDOT provided significant nonlinearity and high dimensionality as a reservoir owing to its intrinsic ion–electron mixed conducting state. Moreover, we investigated the effects of RH on wave generation tasks. Figure 5f displays plots of the accuracy for each waveform as a function of the RH value. As a result, the accuracies for cosine, triangle, and square waves were almost identical with high values (> 90%), irrespective of the RH values. Conversely, the accuracy strongly depended on the RH values for the sawtooth and sin(2ω) waves. Accuracy increased with increasing RH (region (1)), and the highest accuracy was observed in the range of 60–80% RH (region (2)). Afterward, the accuracy decreased again with increasing RH (>80%, region (3)). These trends were consistent with those obtained from impedance spectroscopy measurements. Considering the Fourier series expansion of each waveform, simple waveforms with half‐wave symmetry (cosine, triangle, and square waves) consisted of only odd‐order components, whereas sawtooth and sin(2ω) waves required even‐order components. This finding indicated that a nonlinear transformation from a simple input wave to output waves with a phase shift and asymmetric waveform distortion was required to attain high accuracy for complex waveforms (e.g., sawtooth and sin(2ω)). The frequency response was investigated to obtain further insight into the PR performance, which exhibited a strong dependence on the frequency for the mixed‐conducting state of S‐PEDOT (60% ≤ RH ≤ 80%, Figure S8, Supporting Information). Notably, the accuracy was higher in the frequency region from 10 to 100 Hz. This result was comparable to the time constant (*RC*_eff_) corresponding to the hole (electron) and ion (proton) conduction obtained from the impedance spectroscopy measurements (Table 1). Therefore, we concluded that such a high PR performance could be attributed to the complex dynamics originating from the cooperative hole (electron) and proton mixed conducting state of chemically dedoped S‐PEDOT nanofilms. To our knowledge, these high accuracies are comparable to or better than those reported for other material‐based PR devices, although our device had only 7‐output electrodes (Table S1, Supporting Information).

**Figure 6:**
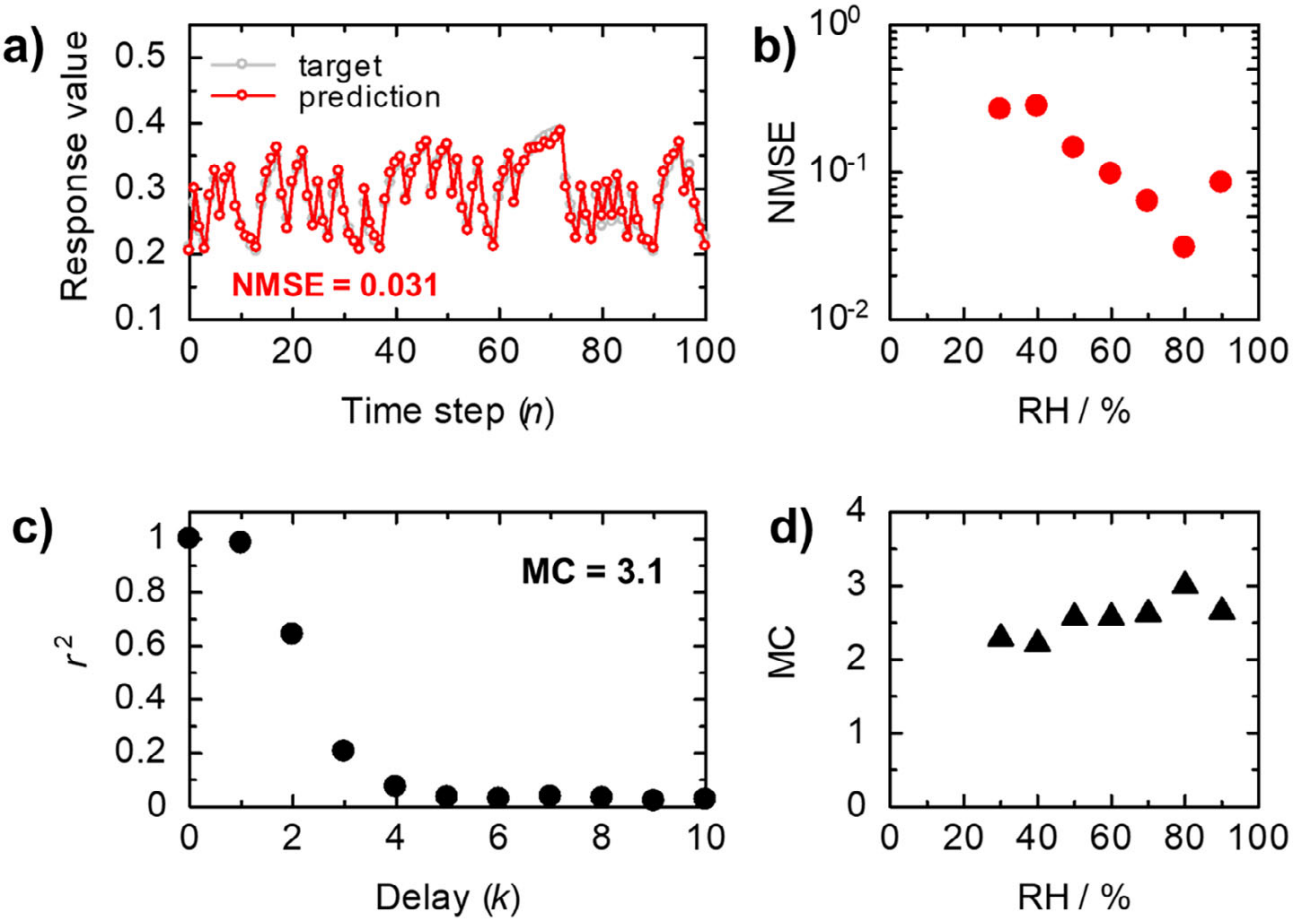
a) Target values (gray) and predicted values (red) of the NARMA2 task measured at RH = 80% and *T* = 25 °C for the dedoped S‐PEDOT nanofilm devices. b) NMSE values calculated from the NARMA2 results under different RH conditions. c) Forgetting curves of the dedoped S‐PEDOT nanofilm devices obtained in the short‐term memory task. d) Plots of MC values as a function of different RH conditions. Triangular random waves (*f* = 100 Hz and V_pp_ = 10 V) were used as input signals in both tasks.

Finally, the ability of the proposed device to solve nonlinear dynamic problems with dedicated S‐PEDOT nanofilm devices was investigated as a typical application of the PR system in spatiotemporal information processing. By using a typical methodology from the literature,^[^ ^18^, ^73^ ^]^ we conducted nonlinear autoregressive moving average (NARMA) 2 tasks as a time series prediction task under controlled RH conditions using ten different waves, where each consists of one thousand triangular pulses with random polarity, with *f* = 100 Hz and V_pp_ = 10 V. The target values in the NARMA2 task were determined by Equation (3):

(3) $$\begin{array}{ccc} y\left(n\right) & = & 0.4y\left(n-1\right)+0.4y\left(n-1\right)d\left(n-2\right)+0.6{\left(\frac{u\left(n-1\right)}{2}\right)}^{3}\\ & & +0.1 \end{array}$$

where *n* is a discrete time step in which each triangular pulse was input, *u*(*n*) and *y*(*n*) are an input and a target at time step *n*, respectively. **Figure** **6**a shows the target and prediction values of the NARMA 2 task for dedoped S‐PEDOT nanofilm devices at RH = 80%. The results revealed good normalized mean squared error (NMSE) values of 0.031. Notably, the NARMA2 task displayed the best performance, with the smallest NMSE value in the ion–electron mixed conducting state as mentioned above (RH = 80%, Figure 6b). Moreover, the memory capacity (MC), calculated from the forgetting curves (Figure 6c) via Equation (4), exhibited a similar tendency (MC = 3.1, RH = 80%).

(4) $$\mathrm{MC}=\sum_{k=1}^{\infty }r^{2}\left(k\right)$$

Here, *r* ^2^ is the determination coefficient at the time step delay (*k*). These results clearly indicated that intrinsically ion–electron mixed conducting states provided significant advantages for in‐material PR devices, suggesting that utilizing multi‐carriers is a new concept for the development of the next generation of material‐based PRs.

## 3 Conclusion

In this study, we demonstrated material‐based PRs of self‐doped S‐PEDOT nanofilms utilizing an intrinsically ion–electron mixed conducting state induced via chemical dedoping approaches. UV–vis–NIR spectroscopy, XPS, and ESR measurements revealed the successful chemical dedoping of the S‐PEDOT nanofilms by TREN. The electrical characteristics of the S‐PEDOT nanofilm were investigated by impedance spectroscopy under controlled temperature and RH conditions. The results revealed that the predominant conducting carriers in the S‐PEDOT nanofilms varied systematically depending on the RH. Under low RH conditions (RH ≤ 50%), the main carriers were holes (*σ*_hole_ >> *σ*_proton_). With increasing RH, the S‐PEDOT film exhibited a mixed conducting state because of its comparable *σ*_hole_ and *σ*_proton_ values under intermediate RH conditions (60% ≤ RH ≤ 80%) (*σ*_hole_ ≈ *σ*_proton_). A further increase in RH resulted in a much higher *σ*_proton_ than *σ*_hole_, indicating that the dominant carriers were protons under high RH conditions (RH > 80%) (*σ*_proton_ >> *σ*_hole_). Moreover, the PR performance of the S‐PEDOT nanofilms is explored by wave generation and NARMA tasks. The S‐PEDOT PRs displayed the best performance at RHs ranging from 60–80% (mixed conducting state). Therefore, we concluded that this high PR performance is attributed to the complex dynamics originating from the cooperative hole–proton mixed conducting state of the S‐PEDOT nanofilms. The high accuracy of S‐PEDOT PR devices is equivalent to or better than that reported for other material‐based PR devices despite having only 7‐output electrodes. The present results suggest that the concept of using multi‐carriers could provide new guidelines for the next generation of material‐based PR devices. Furthermore, by extending this concept to additional ion species such as lithium ions, it holds promise for providing more complex and diverse nonlinear characteristics with various time constants.

## Conflict of Interest

The authors declare no conflict of interest.

## Supporting information



Supporting Information

## Data Availability

The data that support the findings of this study are available from the corresponding author upon reasonable request.
