# Supplementary material for: Utilizing Cooperative Proton–Electron Mixed Conduction Induced via Chemical Dedoping of Self‐Doped Poly(3,4‐ethylenedioxythiophene) Nanofilms for In‐Material Physical Reservoirs
Source: Adv Sci (Weinh). 2025 Dec 18;13(12):e20270. doi: 10.1002/advs.202520270 (PMC12948195; doi:10.1002/advs.202520270)
Supplement: Supplementary file 1 — Supporting Information [file ADVS-13-e20270-s001.docx]

**Supporting Information**

**Utilizing cooperative proton–electron mixed conduction in in-material physical reservoirs of self-doped poly(3,4-ethylenedioxythiophene) nanofilms**

*Yuya Ishizaki-Betchaku,* Motoaki Onishi, Tomoki Misaka, Mitsuo Hara, Hirokazu Yano, Hidenori Okuzaki, Jun Matsui, Tsuyoshi Hasegawa, Takuya Matsumoto, Hirofumi Tanaka, and Shusaku Nagano**

**Experimental section**

**Materials**

All reagents were used without further purification unless otherwise noted. S-PEDOT (SELFTRON^®^(S), 2.1 wt% aqueous solution) was kindly provided by Tosoh Corporation. TREN (96%) was purchased from Sigma Aldrich and used as a dedopant for S-PEDOT. Methanol was purchased from Fujifilm Wako Pure Chemical Corporation. Ultrapure water with a resistivity greater than 18.0 MΩ cm (purified using a Direct-Q 3UV system equipped with a SmartPak water purification system; Merck Millipore) was used as a solvent for S-PEDOT.

**Film preparation**

The quartz substrates were etched using a saturated KOH/ethanol solution under ultrasonication for 15 minutes before use. The substrates were first washed with ultrapure water and then dried under N_2_ gas. Si and Si/SiO_2_ substrates (thermally oxidized SiO_2_ layers ≈ 500 nm) were first washed with CHCl_3_ and then cleaned with oxygen plasma (YHS-R; SAKIGAKE-Semiconductor Co., Ltd.) before use. Gold electrodes with 8 channels, widths of 150 µm and lengths of 30 µm were deposited on each substrate as a blocking electrode using a nickel metal mask by vacuum deposition at < 5×10^–4^ Pa. S-PEDOT nanofilms were prepared by spin-coating using certain concentrations of S-PEDOT aqueous solutions and a spin coater (Opticoat MS-B100; MIKASA) at a spin rate of 2000 rpm for 60 s. The spin-cast films were baked at 200 °C for 30 minutes under vacuum to improve water stability. Afterward, a 1.0 vol% TREN/methanol solution was first spin-coated onto the S-PEDOT nanofilms for dedoping, and then the sample was annealed at 90 °C for 15 minutes to promote the dedoping process.

**Measurements**

TG–DTA was carried out using a DTG-60 system (Shimadzu) under a nitrogen atmosphere at a flow rate of 100 mL min^−1^ controlled by a flow controller (FC-60A). The simultaneous TG–DTA curves were obtained with a heating rate of 10 °C min^−1^ in a temperature range from 30–500 °C by using S-PEDOT flakes of about 7 mg in an aluminum pan. All mass loss percentages were determined using TA-60 WS data analysis software. For DSC measurements of S-PEDOT flakes (~5 mg) in an aluminum pan, Q200 (TA Instruments) was used with a heating and cooling rate of 10 °C min^−1^ under 50 mL min^−1^ of a nitrogen gas flow. The surface morphologies and film thicknesses of the S-PEDOT nanofilms were measured using AFM (SPA400, Seiko Instruments Inc.) with an Al-coated cantilever with a resonant frequency of 111 kHz and a spring constant of 8.7 N m^−1^ (SI-DF-20, Olympus) in AC mode. The electronic state of the S-PEDOT nanofilms was investigated by UV–Vis–NIR absorption spectroscopy using a UV–Vis spectrometer (V-670, Jasco Corp.) with a bandpass of 8.0 nm and a scan speed of 200 nm minute^−1^. FT-IR spectra of S-PEDOT thin films on Si substrates before and after dedoping were obtained using an FT-IR spectrometer (Thermo Scientific Nicolet iS50) equipped with a mercury-cadmium-telluride (MCT) cryo-detector and a spectral resolution of an IR beam of 4 cm^–1^. All the measurements were carried out under a nitrogen atmosphere (AT-10NP-CS). XPS was performed with a spectrometer (ESCA 5700, Ulvac-Phi) featuring a monochromatic Al Kα X-ray source at 298 K under 10^–8^ Torr. The ESR spectra were recorded using a JEOL JES-FA200 spectrometer at room temperature. The film samples, prepared on Si substrates by drop-casting, were cut into small pieces (4 mm × 20 mm) and fixed onto a quartz holder with tape. *In situ* GI-XRS measurements were performed with an X-ray diffractometer (FR-E, Rigaku) equipped with a 2D detector (R-AXIS IV, Rigaku, Tokyo, Japan) under controlled temperature and RH conditions using a precise dew-point generator (me-40DP, Micro Equipment). The electrical conductivity of the S-PEDOT nanofilms was investigated by *I*–*V* measurements using a manual probe station (MBP-55; Apollowave Corp.) equipped with source meters (2612 B and 2450; Keithley Instruments, Inc.). Each data point was collected with a long enough time delay (0.5 s) to measure only a direct current attributed to hole conduction of the S-PEDOT main chains. The impedance spectroscopy measurements were conducted using an inductance, capacitance, and resistance (LCR) meter (IM3536, HIOKI E.E. Corp.) with an applied sinusoidal AC voltage (frequency range = 4 Hz–10 kHz, peak-to-peak voltage (*V*_pp_) = 50 mV). QCM measurements were conducted using a 9 MHz QCM chip with a gold electrode diameter of 5 mm (THQ-100P-SW; TAMADEVICE Co. Ltd.). Time-domain *V*–*t* measurements and wave generation tasks were performed by an arbitrary wave function generator (AWG1005, AS ONE Corp.) equipped with an oscilloscope (Picoscope 5444D MSO, Pico Technology Ltd.). The NARMA2 task was performed using a multifunction generator (WF1947, NF Corp.) equipped with a mixed-signal oscilloscope (MSO44, Tektronix). All the analyses were performed with custom-made Python programs with modules commonly used in our previous literature.^18^ The temperature and RH values were controlled in all electrical measurements using a bench-top-type temperature and humidity chamber (SH-222, ESPEC Corp., Osaka, Japan).

**UV–Vis–NIR spectra**


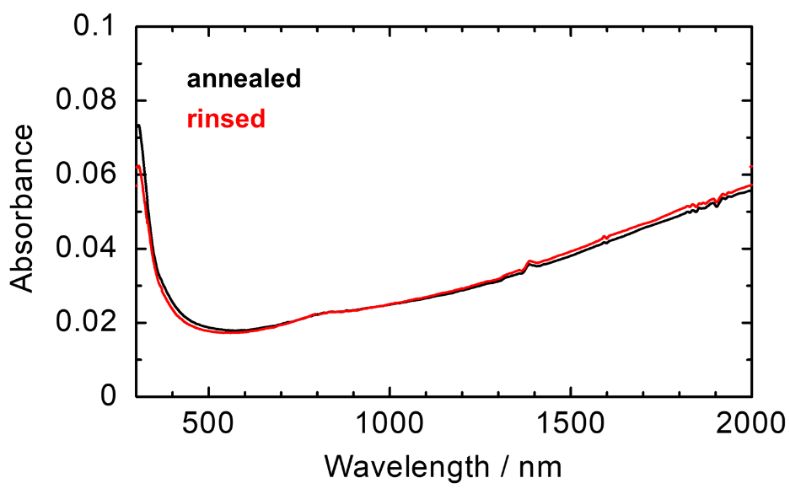


**Figure S1.** UV–Vis–NIR absorption spectra of S-PEDOT at 200 °C for 15 min (black) and after immersing in water for 24 h.

**DG–DTA and DSC curves**

Figure S2 (a) presents the TG–DTA curves of S-PEDOT flakes in the temperature range of 30–500 °C. Three distinct weight-loss regions were identified at (1) 30–150 °C, (2) 200–300 °C, and (3) 300–500 °C, respectively. The first weight loss, accompanied by an endothermic signal, is attributed to the desorption of physically adsorbed water from S-PEDOT. The second endothermic weight-loss process might be associated with non-oxidative partial degradation and cross-linking reactions, involving the cleavage of sulfonate groups in the S-PEDOT side chains.^[S1,S2]^ These two characteristic thermal transitions were also observed in the DSC measurements, as shown in Figure S2 (b). Notably, the endothermic feature appearing at 200–300 °C in the DSC curve emerged only during the second heating cycle (blue line), suggesting the partial cross-linking among the S-PEDOT side chains, which contributes to enhanced thermal stability and improved water resistance. The final pronounced mass loss above 300 °C with an exothermic peak is ascribed to the thermal degradation and carbonization of S-PEDOT.


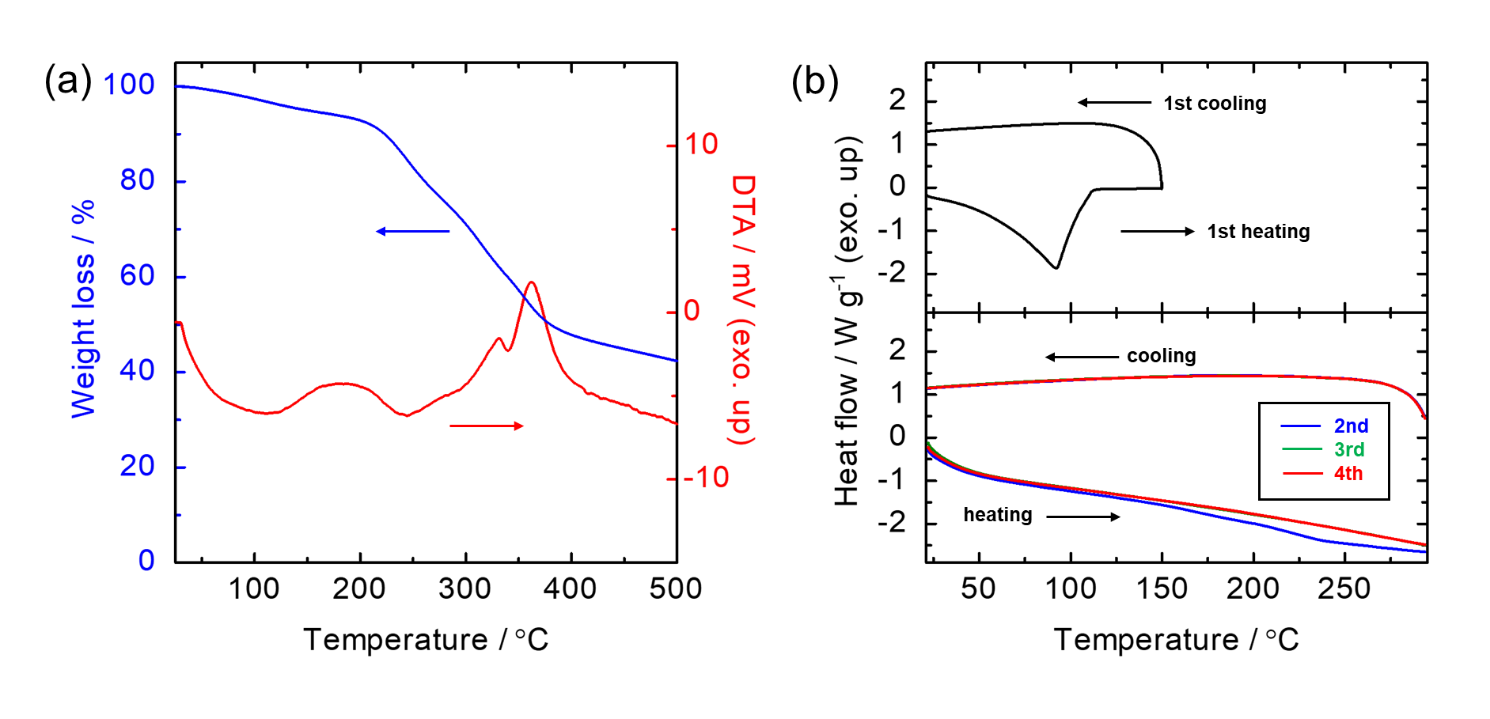


Figure S2. (a) TG (blue)–DTA (red) curves, and (b) DSC traces of S-PEDOT frakes, respectively.

**FT-IR spectra**

Figure S3 displays FT-IR spectra of S-PEDOT nanofilms on Si substrates before (black) and after annealing (blue) and after subsequent dedoping by TREN (red), respectively. Results showed that the relative intensity of the absorption band at 1192 cm^–1^, which originates from O=S=O groups in sulphonate groups,^[S3]^ decreased after annealing. This might reflect partial degradation and cross-linking reactions, involving the cleavage of sulfonate groups in the S-PEDOT side chains. In addition, characteristic vibration modes at 1520 cm^–1^ and 1299 cm^–1^, which are attributable to antisymmetric C*_α_*=C*_β_* stretching and C*_α_*–C*_α_*_´_ inter-ring stretching vibration, were largely shifted to 1508 cm^–1^ and 1319 cm^–1^, respectively, after chemical dedoping. This can be attributed to the structural transformation from the quinoid form to the benzoid structures of S-PEDOT backbones. These results support the partial degradation and crosslinking of –SO_3_H side chains and the successful dedoping of S-PEDOT by TREN.


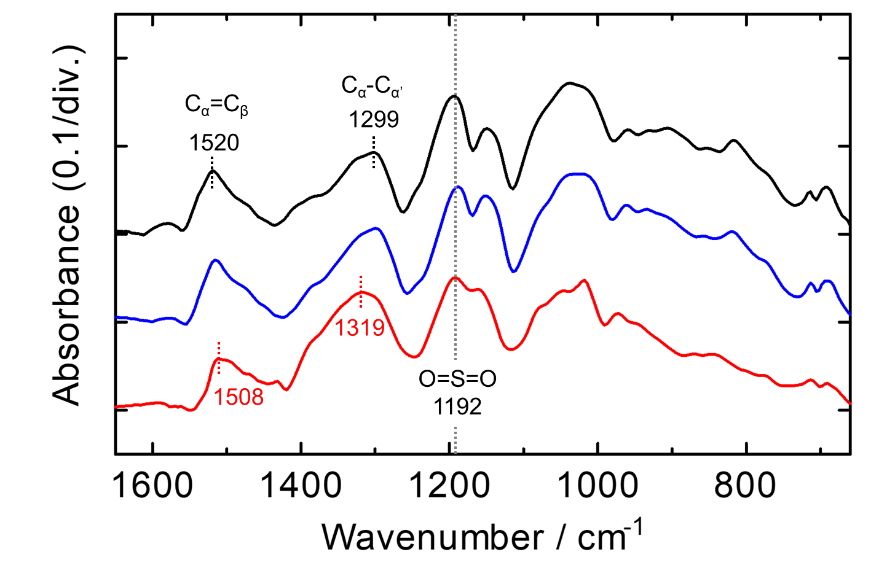


Figure S3. FT-IR spectra of S-PEDOT nanofilms on Si substrates before (black) and after annealing (blue) and after subsequent dedoping by TREN (red).

**ESR spectra**


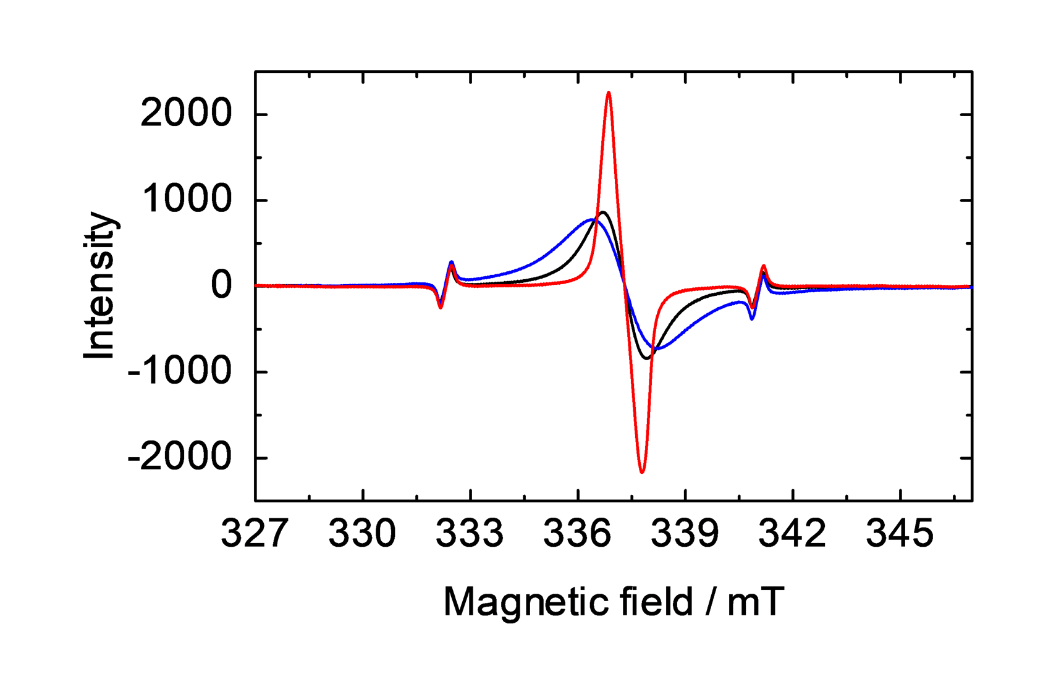


Figure S4. ESR spectra of S-PEDOT nanofilms on Si substrates before (black) and after annealing (blue) and after subsequent dedoping by TREN (red), measured at room temperature.

**Doping/Dedoping process of S-PEDOT**


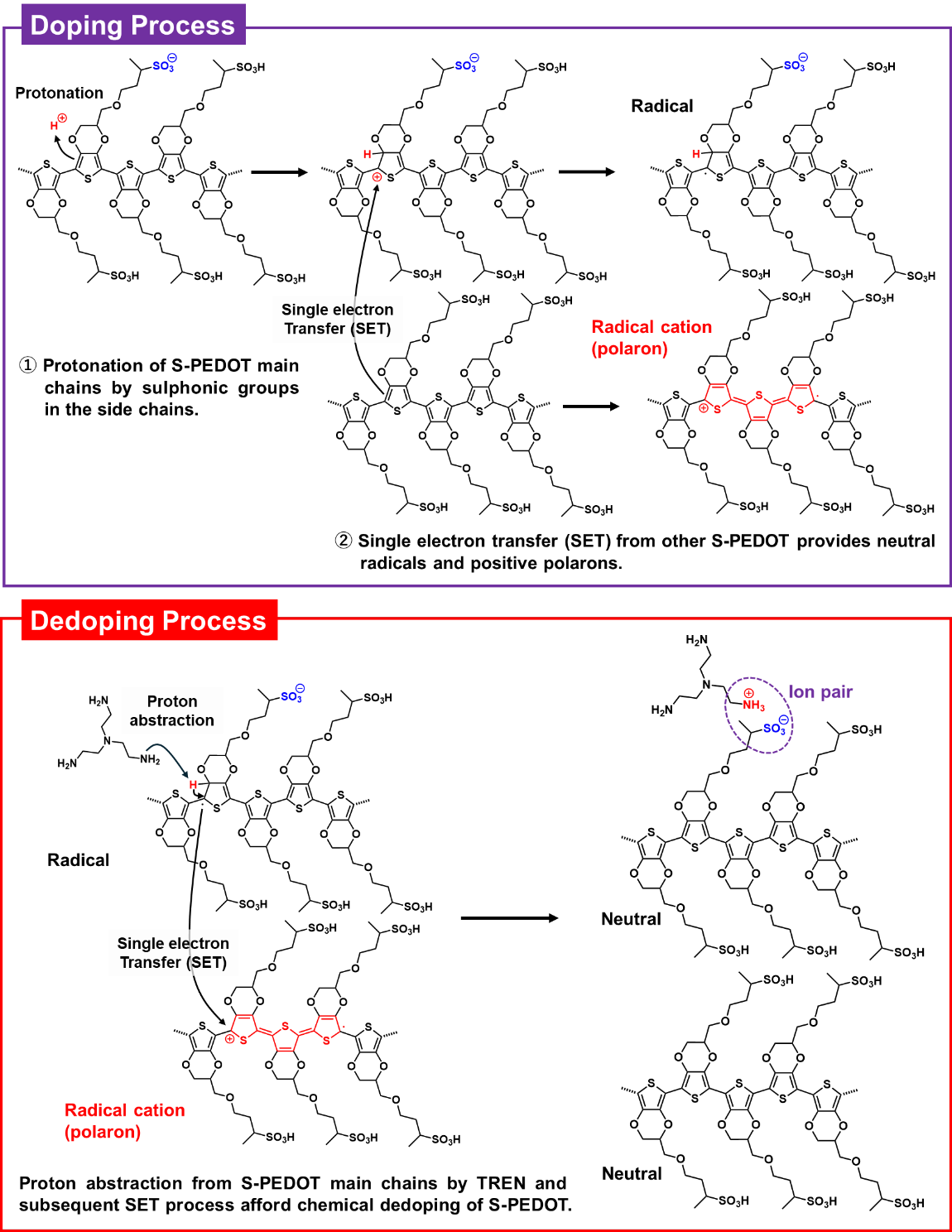


**Figure S5.** Proposed doping and dedoping mechanisms of S-PEDOT.

**AFM images**


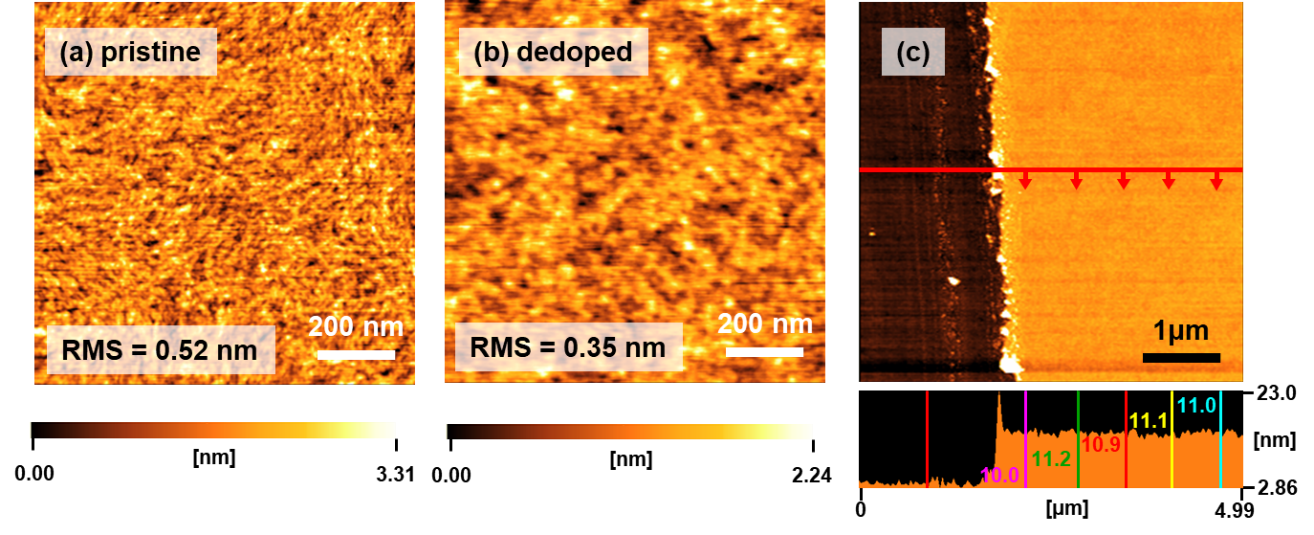


**Figure S6.** Surface morphologies of (a) pristine and (b) dedoped S-PEDOT spin-cast films on Si substrates prepared from 0.25 wt% aqueous solution. (c) The film thickness of (a).

**QCM measurements**


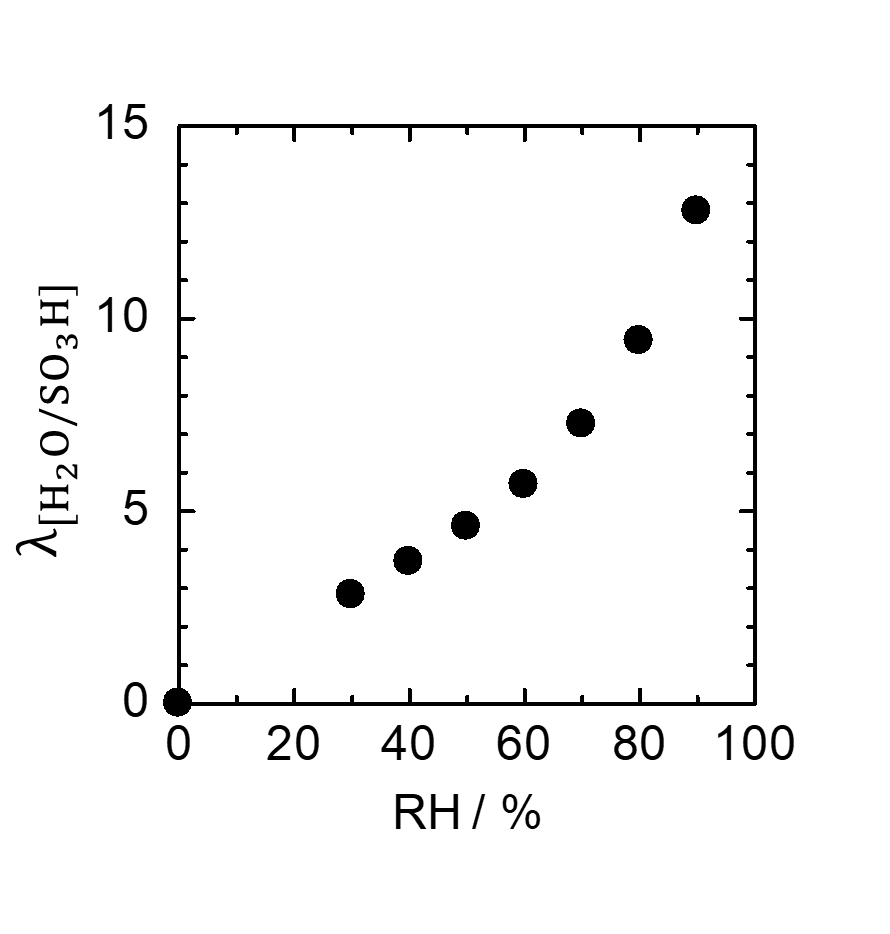


**Figure S7.** Water adsorption of S-PEDOT nanofilms estimated by QCM measurements.

**Wave generation tasks**


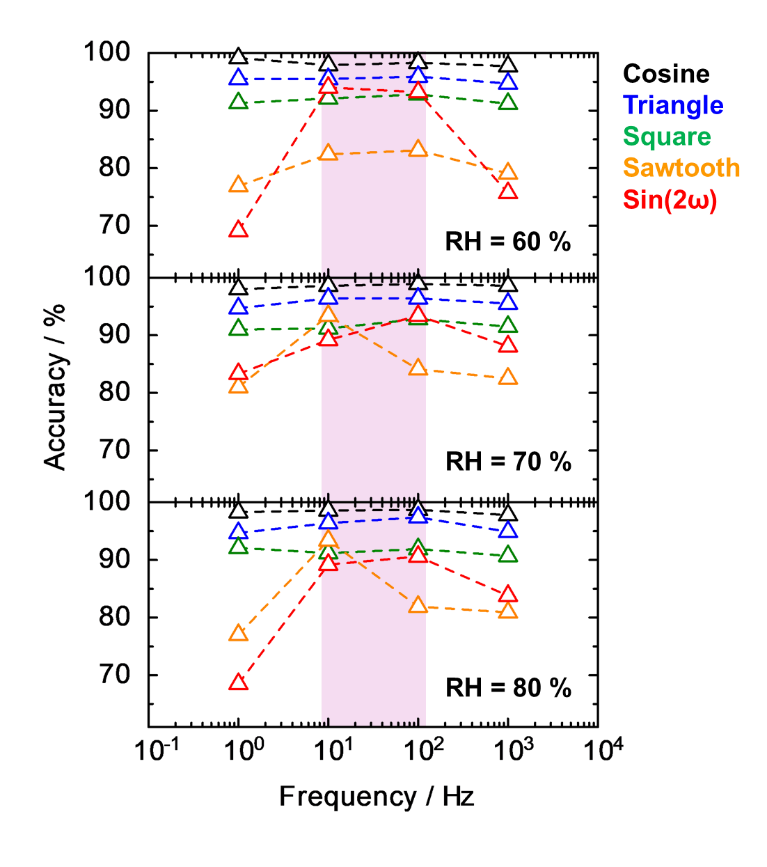


**Figure S8.** Prediction accuracy of waveform generation tasks for dedoped S-PEDOT nanofilms using an input sinusoidal wave with different frequencies under different RH conditions: RH = 60% (top), 70% (middle), and 80% (bottom), respectively.

**Table S1.** Comparison of performance in waveform generation tasks for various physical reservoir devices.

| Materials | | Number of electrodes | Prediction accuracy / % | | | | | Refs |
| --- | --- | --- | --- | --- | --- | --- | --- | --- |
|  |  |  | Cosine | Square | Triangle | sawtooth | Sin(2ω) |  |
| Ag nanowire | 128 | | 90.3 | 73.4 | 90.1 | 89.8 | – | 6 |
| Ag/Ag_2_S core–shell NPs | 15 | | 99.9 | 87.5 | 98.3 | 70.2 | – | 13 |
| SWCN/Por-POM complex | 11 | | 99.0 | 87.0 | 99.4 | 71.0 | – | 15 |
| organic molecules with nanorod/NPs | 88 | | – | 95.1 | 97.7 | – | 97.1 | 19 |
| Conducting polymer | 15 | | 99 | 91 | – | 88 | 93 | 25 |
| Conducting polymer | 15 | | ~99 | ~85 | ~99 | ~70 | ~55 | 27 |
| Mixed conducting polymer | 7 | | 98.6 | 91.2 | 96.4 | 93.4 | 89.2 | This work |

References

[S1] J. L. Carter, C. A. Kelly, M. J. Jenkin, *Polym. J*. **2023**, *55*, 253–260.

[S2] B. Friedel, P. E. Keivanidis, T. J. K. Brenner, A. Abrusci, C. R. McNeill, R. H. Friend, N. C. Greenham, *Macromolecules* **2009**, *42*, 6741–6747.

[S3] O. Y. Posudievsky, N. V. Konoshchuk, A. G. Shkavro, V. G. Koshechko, V. D. Pokhodenko, *Synth. Met.* **2014**, *195*, 335­–339.

[S4] Q. Weinbach, N. Hmili, E. Gottis, G. Fleith, J. Combet, V. Papaefthimiou, V. Malesys, E. Denys, L. Simon, M. Schmutz, A. Carvalho, D. Constantin, L. Biniek, *J. Mater. Chem. C* **2023**, *11*, 7802–7816.

[S5] Z. U. Khan, O. Bubnova, M. J. Jafari, R. Brooke, X. Liu, R. Gabrielsson, T. Ederth, D. R. Evans, J. W. Andreasen, M. Fahlmanc, X. Crispin, *J. Mater. Chem. C* **2015**, *3*, 10616–10623.
